# Cancer Stem Cells or Tumor Survival Cells?

**DOI:** 10.1089/scd.2018.0129

**Published:** 2018-10-25

**Authors:** Yang D. Teng, Lei Wang, Serdar Kabatas, Henning Ulrich, Ross D. Zafonte

**Affiliations:** ^1^ Department of Physical Medicine and Rehabilitation, Harvard Medical School and Spaulding Rehabilitation Hospital Network, Brigham and Women's Hospital, and Massachusetts General Hospital, Boston, Massachusetts.; ^2^ Department of Neurosurgery, Harvard Medical School, Boston, Massachusetts.; ^3^ Division of SCI Research, VA Boston Healthcare System, Boston, Massachusetts.; ^4^ Departamento de Bioquímica, Instituto de Química, Universidade de São Paulo, São Paulo, Brazil.

**Keywords:** cancer stem cell, tumor survival cell, cancer cell survivology, dedifferentiation, stem cell, stemness

## Abstract

Research endeavors originally generated stem cell definitions for the purpose of describing normally sustainable developmental and tissue turnover processes in various species, including humans. The notion of investigating cells that possess a vague capacity of “stamm (phylum)” can be traced back to the late 19th century, mainly concentrating on cells that could produce the germline or the entire blood system. Lately, such undertakings have been recapitulated for oncogenesis, tumor growth, and cancer cell resistance to oncolytic therapies. However, due to the complexity and basic life-origin mechanisms comprising the genetic and epigenetic repertoire of the stemness in every developing or growing cell, presently there are ongoing debates regarding the biological essentials of the stem cell-like tumor initiation cells (ie, cancer stem cells; CSCs). This conceptual analysis focuses on the potential pitfalls of extrapolating that CSCs bear major traits of stemness. We propose a novel nomenclature of *Tumor Survival Cells* (TSCs) to further define tumor cells behaving like CSCs, based on the ruthless and detrimental features of *Cancer Cell Survivology* that appears fundamentally different from stem cell biology. Hence, precise academic separation of TSCs from all the stem cell-related labels applied to these unique tumor cells may help to improve scientific reasoning and strategies to decode the desperado-like survival behaviors of TSCs to eventually overcome cancer.

## Background

Ernst Haeckel (1834–1919), a German biologist, physician, naturalist, artist, and philosopher, is considered a pioneer in developmental cell biology research. He proposed the nomenclature of “Stammzellen” (stem cells) in his published lectures on “Natürliche Schöpfungsgeschichte” (1868) for unicellular organisms or protozoa that he thought to be the phylogenetic ancestors of multicellular organisms [1]. He considered that the name stem cell seemed to be the most explicit and appropriate one for a pluripotent cell phenotype, from which all other cells stem. They are, in the most literal sense, the stem father and the stem mother of all the infinitive generations of cells that the multicellular organism is ultimately built with [1]. The term was created to distinguish the unique profile of the fertilized egg cell from the original egg cell. Following the doctrine, the human stem cell directly represented the whole future child [2,3].

Haeckel's neologism was derived from the metaphorical language popularly used back then in medical and philosophical discussions about cells and the human body. Scholars (eg, Rudolf Virchow: 1821–1902) often compared cells in a given organism with human individuals within an established state system [4]. For the first time, the concept of a stem cell defined cell state in a hierarchical and centralized format, departing from the previous conception of a liberal and relatively egalitarian profile. It is worth noticing that (1) stem cells are primordial biological entities destined to build a homeostatic system that can reproduce itself, and (2) the metaphorical implication of the stem cell concept not only has its general public education value, but more importantly, its usage deeply impacts the way scientists frame and orient their reasoning.

For example, at the turn of the 19th century, Artur Pappenheim (1870–1916), Alexander Maximow (1874–1928), Ernst Neumann (1798–1895), and other scholars proposed a progenitor cell-based theory for the common origin of all hematopoietic lineages. In the beginning of the 20th century, the advancements in the field of hematopoiesis and leukemia research further distinguished the stem cell definition, underscoring *a common central capacity for self-renewal and phenotypic differentiation* [2].

## The Evolving Theory of Cancer Stem Cells

Possible underlying relations between embryonic stem cells (ESCs) and normal tissue or cancer-like neoplasm were also speculated in the late 19th century. The notion concerned the chances for development deviations of ESCs to contribute to malformation or tumorigenesis [5]. However, key components of this tumorigenic theory (eg, the displacement of embryonic cells) were questioned by gathering experimental evidence around World War II [6]. In the 1950s and early 1960s, systematical investigation of murine teratoma cells resulted in successful isolation of mouse ESCs and basic characterization techniques. The research progression further cultivated the postulation of existence of the so called cancer stem cells (CSCs) [7]. By the early 1980s, murine ESCs could be reliably isolated and maintained in vitro [8,9], which, together with the identification of human neural stem cell and human ESC lines laid down the foundation for opening the contemporary chapter of stem cell research [10–12].

In parallel, the concept of CSCs was gradually shaped out in the 1960s. For instance, Kleinsmith and Pierce demonstrated that donor embryonal carcinoma cells (ECCs) could give rise to both somatic tissue cells and ECCs [7]. It was reported that only ∼0.1%–1% of murine myeloma cells could give rise to new clones in vitro, and only ∼1%–4% of leukemia cells formed macroscopic colonies in the spleen after transplantation in nonobese diabetic/SCID (severe combined immune deficiency) mice [13]. Noticeably, the data showed certain similarities with the formation of nodules that was observed in the spleens of irradiated mice following administration of bone marrow cells. The number of nodules generated was found to be dose dependent on the quantity of the injected bone marrow cells. Thus, the investigators hypothesized that a single hematopoietic stem cell (ie, colony-forming unit) might be able to develop into a cell colony that gradually formed an individual nodule [14]. These findings combinatorially inferred the possibility that a limited number of tumor cells might have “stem cell-like” oncological behavior and act as a ringleader for tumor initiation. Taken together, these discoveries promoted the establishment of the CSC theory.

By the mid-1970s, the clonal evolution theory of cancer growth was additionally enriched by uncovering that mutations in oncogenes and tumor suppressor genes played important roles in tumorigenesis [15]. Fearon and Vogelstein proposed that the stepwise acquisition of mutations in specific oncogenes was critical in the progression and malignization of early adenoma, based on their clonal evolution model of colon cancer [16]. The feature of colon cancers indeed exhibited a generally linear tumor evolution with incremental genetic mutations following inactivation of adenomatous polyposis coli as the most common gene mutation. Elucidating these genetic mechanisms helped to address the question of why a given malignant tumor lesion may contain a subpopulation of cells that show everescalating malignant behavior [16]. By contrast, breast cancers retain discernible levels of intratumoral heterogeneity [17]: for example, amplification of HER2 (human epidermal growth factor receptor 2), mutation of PIK3CA (phosphoinositide-3-kinase, catalytic, alpha polypeptide), etc. Moreover, similar heterogeneity exists in leukemia. Nearly all subtypes of acute myeloid leukemia (AML) can be implanted in immunodeficient mice by engraftment of a CD34^+^CD38^−^ fraction of AML cells (ie, acute myelogenous leukemia stem cells, LSCs: ∼1/million AML cells) [18].

At the beginning of the 21st century, the concept of CSC or tumor stem cell was refined based on the evidence that certain developmental signaling pathways governing regular stem cells might also function in CSCs for tumor formation [19]. Therefore, CSCs, as a small subpopulation of tumor cells, were proposed as the primary force fueling oncogenesis and were characterized by their capability of infinite self-renewal and drug resistance [19]. For example, to initiate a new tumor in a mouse, only ∼100 CD44^+^CD24^−/low^ human breast cancer cells are required, indicating their potent cancerogenic potential relative to other phenotypes of tumor cells that fail to grow tumors even under thousandfold higher quantity [20]. The consensus definition of a CSC was first established by the participants of the 2006 American Association of Cancer Research Workshop on Cancer Stem Cells. The definition states that CSCs should possess the properties of tumorigenicity, self-renewal capacity, continuous passage ability, multilineage differentiation potential to generate the heterogeneous subpopulations of cancer cells that comprise malignant tumors, and unique and reliable surface markers [20–23].

## Reciprocal Interaction Between CSC and Normal Stem Cell Research Studies

The CSC theory, besides its academic impact, has practically revealed new therapeutic targets for designing specific therapies to treat cancer. To this end, knowledge gleaned from investigating normal stem cells has markedly improved the understanding of the heterogeneous nature of cancer cells [19,23]. It is believed that CSCs hold higher oncologic plasticity than regular cancer cells; this plasticity may be powered mainly by stemness-like capabilities, promoting efforts to identify key triggers of neoplasm occurrence/recurrence, metastasis, and drug resistance [19–25]. Therefore, it is pivotal to investigate whether or to which scale CSCs may genetically overlap with normal stem cells. Ultimate understanding of these mechanisms will facilitate therapeutic development for managing cancer.

Indeed, a subgroup of CSCs has been found to behave as tumor metastasis and recurrence (or drug resistance)-initiating cells due to their quiescent state, tumorigenicity, and migration capabilities. The cells express markers of epithelial mesenchymal transition (EMT), including collagen IV α1, α-SMA, β-catenin, etc. [24]. EMT describes the process of the transdifferentiation of stationary epithelial cells into motile mesenchymal cells. Over EMT evolvement, epithelial cells lose their tight junctions and apical–basal polarity, reorganize their cytoskeleton, and experience changes in the signaling cascades that control cellular morphological features and gene expression programs. These alterations share main features of *dedifferentiation*, increasing the motility of individual cells, and enabling them to develop into phenotypes with invasive behaviors that are crucial for *cell survival*. EMT process can be regulated or influenced by multiple pathways that are activated by TGF-β, HGF, EGF, FGF, VEGF, Wnt, Shh, IL6, HIF1α, and other signaling molecules through SNAI1/Snail, ZEB1/ZEB2, and/or basic helix-loop-helix proteins-mediated transcription activity [25]. EMT events are crucial in major biological courses of embryonic development, postnatal growth, tissue regeneration, lesion healing, and stem cell homeostasis. EMT-like mechanisms have also been implicated in triggering oncology of malignancies and pathophysiology of fibrosis [25]. The involvement of dedifferentiation as a stem cell-like feature (ie, stemness) in some cancer cells to drive tumorigenesis has been further validated by new findings published in The Pan-Cancer Atlas, the official data portal of The Cancer Genome Atlas (TCGA) consortium. Specifically, TCGA tumors' (ie, 11,000 tumors from 33 of the most prevalent forms of cancer) epigenetic and expression-based stemness indices measured oncogenic dedifferentiation and revealed association with oncogenic dedifferentiation. The investigators reported that the dedifferentiated oncogenic phenotype was generally most prominent in metastatic tumors. The indices identified novel targets for designing potential therapies to augment tumor differentiation [26]. Importantly, it has been recognized that dedifferentiation is likely a mechanism for cell survival [27]. We, therefore, suggest to also focus on survival benefits that can be derived from the intratumor molecular heterogeneity determined by the stemness indices reported [26], to dissect it from classically defined stemness indices of developmental biology that emphasize differentiation [28].

## CSCs Do Not Possess Authentic Stemness Biology

Researchers have determined a variety of surface markers for identifying CSCs. As examples, currently well-accepted markers for glioma CSCs include CD15, CD90, CD133, nestin, and integrin-α6. CD44, ALDH, CD117, CD133, and CD24 are utilized to profile ovarian CSCs. For malignant melanoma CSCs, ABCB5, ALDH1, CD20, CD133, and CD271 are commonly enlisted. ALDH1, CD44, CD24, CD90, and CD133 are highlighted as markers for breast CSCs. There are some shared CSC markers frequently expressed in different types of malignant tumors. Among them, CD133 appears to be the most common one, which coincidently is also a marker of normal stem cells (eg, undifferentiated ESCs, HSCs, and NSCs).

**FIG. 1.**
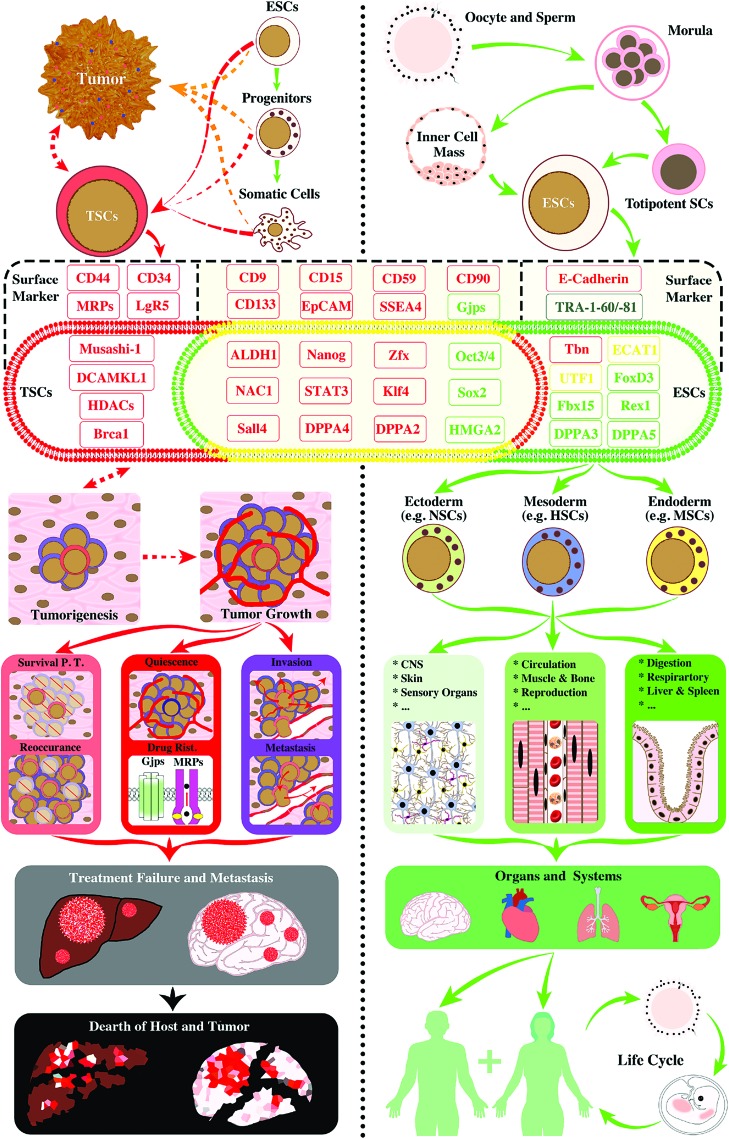
Established molecular markers of TSCs (also called CSCs; *left panel*) and ESCs (*right panel*). In support to our hypothesis, TSCs possess genes either uniquely to themselves (markers in *red zone* and *red font*) or shared with ESCs (molecules in *yellow zone*) that are predominantly related to cell survival functions (eg, proliferation, migration, invasive growth, drug resistance, etc.; markers in *red fonts*). They play critical roles in cancer metastasis, reoccurrence, and oncolytic treatment failure. Although TSCs in different types of malignant tumors share numerous molecular markers with ESCs (markers in *yellow zone*), they are deficient in molecules that are essential for the maintenance of pluripotent status, self-renew, and lineage-specific differentiation, key features of authentic stemness for physiological development and reproduction of biological organisms/entities, including humans (markers in *green fonts*). This unbalanced desperado-like survival strategy of TSCs disrupts homeostasis and exhausts resources essential for host life, which inevitably leads to demises of both host and tumor cells (*left panel* flowcharts). By contrast, totipotent stem cells, inner cell mass-derived ESCs, carry stemness genes mostly for physiological development (markers in *green font* and *green zone*) and keep an effective balance between cell development (markers in *green font*), tissue formation (eg, markers in *yellow*: for cell differentiation), and survival (markers in *red font*; note: functions of the marker molecule in *black font* are presently unclear). These genes work in consortium to drive proper cell proliferation and migration, lineage differentiation, organ genesis and systemic homeostasis, and to make the biological species sustainable. For example, human ESCs differentiate into progenitor cells of the three primary germ layers that subsequently establish functional tissues, organs, and systems. With a new embryo implantation and growth, the whole process of ESC-originated development results in a sustainable life circle for human race (*right panel* flowcharts). The process defines the authentic stemness capacity (ie, stamm or phylum). CSC, cancer stem cell; Drug Resi., drug resistance; ESC, embryonic stem cell; Gpj, gap junction; HSC, hematopoietic stem cell; MDR, multiple drug resistance; NSC, neural stem cell; PT, posttreatment; TSC, tumor survival cell. Color images available online at www.liebertpub.com/scd

**FIG. 2.**
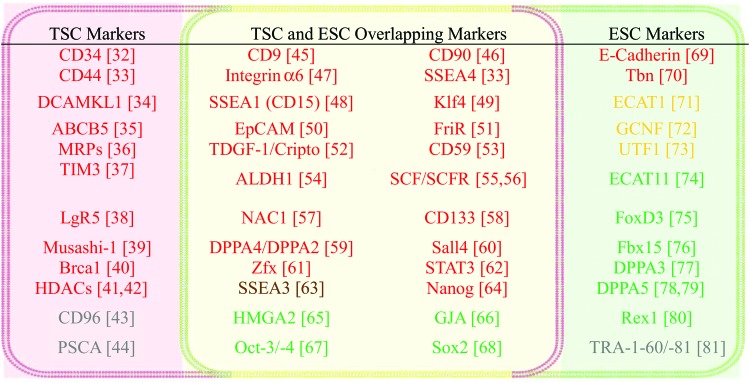
Common molecular markers of TSCs and ESCs. Color codes: (1) *Green*: genes related to self-renewal and maintenance of pluripotent status or dedifferentiation; (2) *Yellow*: genes important for cell differentiation; (3) *Brown*: genes that support cell survival and cell proliferation but are not essential for the maintenance of pluripotent status of cells; (4) *Red*: genes enabling cancer cell migration, metastasis and invasion, and/or drug resistance; (5) *Gray*: specific genetic markers that are presently unclear for their functions. Color images available online at www.liebertpub.com/scd

However, in spite of accumulation of published data that is in favor of the CSC concept, the validity of the stemness biology in CSCs has been constantly challenged by observations concerning discrepancies regarding the biological characteristic, phenotype, genetic profile, and subpopulation proportion ratio of the alleged “original” CSC. Studies showed that successful isolation rate of glioma CSCs was dependent on microenvironmental specifics, including cell–cell interaction, culture medium composition, and cell incubation temperature [29,30]. The data begin to contest the existing CSC theory and arguably suggest that these cells could be a reactive dedifferentiation consequence of regular cancer cells driven by microenvironmental stress, attempting to maximize the survival probability of the tumor, rather than an outcome of a conventionally defined hierarchical cascade of tumor cell development. In fact, CSC-produced intratumor heterogeneity is incapable to form any truly sustainable biological system such as normal tissues or organs. The dedifferentiated tumor cells seemed to be destined to refill the pool of previous CSCs upon their depletion resulting from regular cancer cell differentiation, host immune counteraction, and/or anticancer treatment [31]. Thereby, we *hypothesized* that the commonly defined CSCs might mostly retain genes underlying cell survival (eg, dedifferentiation, proliferation/self-renewal, migration, engraftment, and drug resistance) relative to those of normal stem cells that fundamentally concern lineage-oriented differentiation, homeostasis, stemness, and sustainability through self-renewal and reproduction (Figs. 1 and 2 and Table 1). To test this hypothesis, we systematically examined a total of 50 established molecular markers of CSCs and/or ESCs. The results demonstrated that (1) CSC exclusive markers are genes that support cancer cell migration, metastasis and invasion, and/or enable drug resistance capability (see Figs. 1 and 2 for red markers) [32–42], and only two of them bear uncertain functions (gray markers: CD96 [43] and PSCA [44]; Fig. 2); (2) the majority of markers shared by CSCs and ESCs are genes that are also related to cell migration and metastasis or engraftment (see Figs. 1 and 2 for red markers [42–59,61]) [45–63], with one related to proliferation but not stemness (brown marker: stage-specific embryonic antigen-3; SSEA3; Fig. 2) [64], and a few concerning self-renewal and maintenance of pluripotent status or dedifferentiation (green markers: HMGA2 [65], GJA [66], Oct-3/-4 [67], and Sox [68]; Fig. 2); and (3) notably, of markers selective for ESCs, there are two molecules for cell migration and engraftment (red markers; Fig. 2) [69,70], three factors important for cell differentiation (yellow markers; Fig. 2) [71–73], and mostly, genes enabling self-renewal and maintenance of pluripotent status or lineage differentiation (green markers; Fig. 2) [74–80], except for one gene with unclear function (gray marker: TRA-1-60/-81; Fig. 2) [81].

**Table 1.** Common Molecular Markers of Tumor Survival Cells and Embryonic Stem Cells and Their Functions

*Abbreviation*	*Full name*	*Subcellular location*	*Function*	*Refs.*
TSC-specific markers				
CD34	CD34	Cell surface	Cell adhesion and migration	[32]
CD44	CD44	Cell surface	Cell adhesion, migration and metastasis	[33]
DCAMKL1	Doublecortin like kinase 1	Cytoplasm	Epithelial–mesenchymal transition, cancer invasion and metastasis	[34]
ABCB5	ATP-binding cassette subfamily B member 5	Cell surface	Drug resistance	[35]
MRPs	Multidrug resistance pumps	Cell surface	Drug resistance	[36]
TIM3	T cell immunoglobulin and mucin domain 3	Cell surface	Drug resistance, tumorigenesis, self-renewal in leukemic stem cells	[37]
LgR5	Leucine-rich repeat-containing G-protein-coupled receptor 5	Cell surface	WNT signaling and related cancer metastasis	[38]
Musashi-1	RNA-binding protein Musashi homolog 1	Nucleus and cytoplasm	Posttranscriptional regulation of self-renewal and differentiation	[39]
Brca1	Breast cancer 1	Nucleus	DNA repair of double-strand breaks and mismatch	[40]
HDACs	Histone deacetylases	Nucleus	Histone modification, drug resistance, cell proliferation, and growth	[41,42]
CD96	Tactile	Cell surface	Cell adhesive interaction and specific TSCs marker	[43]
PSCA	Prostate stem cell antigen	Cell surface	TSC-specific marker	[44]
Overlapping markers				
CD9	CD9 antigen	Cell surface	Cell adhesion, migration, and regulation of cell development	[45]
CD90	Thy-1 cell surface antigen	Cell surface	Cell adhesion, migration, and metastasis	[46]
Integrin α6	Integrin alpha 6	Cell surface	Cell adhesion, differentiation, polarity, proliferation, survival/apoptosis	[47]
SSEA4	Stage-specific embryonic antigen 4&1	Cell surface	Cell adhesion and migration	[33,48]
SSEA1				
Klf4	Kruppel-like factor 1	Nucleus and cytoplasm	Tumor migration, invasion, and ESCs self-renewal	[49]
EpCAM	Epithelial cell adhesion molecule	Cell surface	Cell adhesion, and WNT signaling	[50]
FriR	Frizzled receptors	Cell surface	WNT signaling receptors, related to cell proliferation, migration, and survival.	[51]
TDGF1/Cripto	Teratocarcinoma-derived growth factor 1	Cell surface and cytoplasm	Tumor anchorage-independent growth and proliferation	[52]
CD59	CD59	Cell surface	Cell survival and inhibit homologous complement-mediated cytolysis	[53]
ALDH1	Aldehyde dehydrogenase 1	Cytoplasm	Retinoid metabolism and self-renewal, cell proliferation, drug resistance	54]
SCF	Stem cell factor	Cytoplasm	Drug resistance, cell migration and stemness	[55,56]
SCFR	Mast/stem cell growth factor receptor, CD117	Cell surface	Drug resistance, cell migration and stemness	[55,56]
NAC1	Nucleus accumbens-associated protein1	Nucleus	Drug resistance and ESCs self-renewal	[57]
CD133	CD133	Cell surface and cytoplasm	Cell proliferation and dedifferentiation	[58]
DPPA2/4	Developmental pluripotency-associated 2/4	Nucleus	Tumor cell initiation, proliferation and ESCs maintenance of pluripotency	[59]
Sall4	Spalt-like transcription factor 4	Nucleus	Cell proliferation, drug resistance, and ESCs self-renewal	[60]
Zfx	Zinc finger protein X-linked	Nucleus	Cell proliferation	[61]
STAT3	Signal transducer and activator of transcription 3	Cytoplasm and nucleus	Tumor cell proliferation, survival, invasion, and ESCs self-renewal	[62]
SSEA3	Stage-specific embryonic antigen 3	Cell surface	Cell survival and cell proliferation but not necessary for maintenance of pluripotent status	[63]
Nanog	Homebox protein nanog	Nucleus	Self-renewal, maintenance of pluripotency, and drug resistance	[64]
HMGA2	High-mobility group AT-hook 2	Nucleus	Self-renewal and differentiation	[65]
GJA	Gap junction protein	Cell surface	Self-renewal and intercellular communication	[66]
Oct-3/-4	Octamer-binding transcription factor 3/4	Nucleus	Dedifferentiation and ESCs self-renewal	[67]
Sox2	(Sex-determining region Y)-box 2	Nucleus	Tumor initiation and ESCs self-renewal	[68]
ESC-specific markers				
E-Cadherin	E-Cadherin	Cell surface	Cell adhesion, migration, and pluripotency	[69]
Tbn	Taube nuss	Nucleus	Cell survival, regulating the extent of programmed cell death	[70]
ECAT1	ES cell associated transcript 1	Nucleus	Oocyte maturation and preimplantation development	[71]
GCNF	Germ cell nuclear factor	Nucleus	Differentiation	[72]
UTF1	Undifferentiated embryonic cell transcription factor 1	Nucleus	ESCs self-renewal and differentiation	[73]
ECAT11	ES cell-associated transcript 11	Nucleus	ESCs self-renewal	[74]
FoxD3	Forkhead box protein D3	Nucleus	ESCs self-renewal	[75]
Fbx15	F-box-only protein	Nucleus	ESCs self-renewal	[76]
DPPA3/5	Developmental pluripotency-associated 3/5	Nucleus	Acquisition and maintenance of pluripotency	[77–79]
Rex1	Reduced expression 1	Nucleus	Acquisition and maintenance of pluripotency	[80]
TRA-1-60/80	Podocalyxin-like protein 1	Cell surface	ESC-specific marker	[81]

Therefore, the analytical outcome in general confirms our postulation. Although CSCs share many genetic markers with ESCs, deeper dissection revealed that CSC-specific genes are predominantly in charge of cell survival activities typically involving invasive growth, cell migration, and survival adaptation (eg, intratumoral heterogeneity and drug resistance), which jointly play critical roles in cancer metastasis, reoccurrence, and insensitivity to chemotherapy and host immune counteractions. By contrast, inner cell mass-derived ESCs exhibit a balanced profile between genes responsible for authentic stemness maintenance emphasizing pluripotency, self-renewal, capability of lineage-specific differentiation and development into reproducible organisms, and genes empowering cell homeostatic survival (Fig. 1; see detailed information in Table 1). Evidently, the biological trajectories of CSCs, which are hallmarked by unilateral attempts for self-survival at the expense of regular cancer and host cells, do not match natural paradigms of developmental biology-related stem cells. Normal stem cells, alongside their proliferation, migration, and differentiation, constantly build homeostasis with surrounding cells through their functional multipotency [82]; and once differentiated into terminal phenotypes, they will not dedifferentiate under physiological conditions.

In corroboration with our analysis, published experimental results clearly demonstrate that all colonies derived from randomly selected single cells of murine lung and breast cancer cell lines can form tumors following allografting in histocompatible mice [83]. A recent study reported that using an approach that integrated major immunogenomics methods (ie, total lymphocytic infiltrate assessed from genomic and haemotoxylin and eosin staining image data, immune cell fractions from deconvolution analysis of mRNA-sequencing data, immune gene expression signatures, neoantigen prediction, T cell receptor and B cell receptor repertoire inference, viral RNA expression, and somatic DNA alterations) to characterize the immune tumor microenvironment (TME) (ie, the immune subtype), investigators identified six immune subtypes that span TCGA cancer tissue types and molecular subtypes [84]. Cancer immune subtypes differ by somatic aberrations, TME, and survival, but not by differentiation. The six immune subtypes are wound healing, IFN-γ dominant, inflammatory, lymphocyte depleted, immunologically quiet, and TGF-β dominant, all being characterized by differences in macrophage or lymphocyte signatures, Th1:Th2 cell ratio, extent of intratumoral heterogeneity, aneuploidy, extent of neoantigen load, overall cell proliferation, expression of immunomodulatory genes, and prognosis [84]. Again, the heterogeneous features of tumor–immune cell interactions are mechanisms underlying cell survival, not sustainable development and organ genesis [85]. Interestingly, TME by definition contains the anatomically distinct regions defined as CSC niches that maintain CSCs by preserving their self-renewal, clonal tumor initiation capacity, and clonal long-term repopulation and metastatic potential, as well as by shielding them from immune surveillance [86]. It has been shown that cells within the CSC niches produce factors that stimulate CSC self-renewal, induce angiogenesis, regulate immune cells, and recruit other stromal cells that secrete additional factors to promote tumor cell survival (eg, invasion and metastasis), Conversely, cells composing normal stem cell niches (eg, neural stem cell niches) affect stem cell differentiation in addition to preserving their self-renewal through numerous biophysical and biochemical mechanisms [87]. Presentation of systematical comparisons between CSC niches and those of regular stem cells is beyond the scope of the current work; however, such analytical outcomes will undoubtedly help us to better understand the fundamental biology of CSCs. The data have kept kindling our intention to suggest that the fundamental developmental biology principles should caution application of the stem cell concept in labeling any terminal oncological and pathological cell phenomena [83].

## Present Definitions of CSCs

To substantiate our proposal of defining an alternative term for CSCs, we have analyzed the following definitions currently used to describe CSCs [88].

1. CSCs may directly derive from normal stem cells through genetic mutation. Thus, these cells have the ability for self-renewal and differentiation into all heterogeneous tumor cell phenotypes of a particular cancer (note: intratumoral heterogeneity maximizes cancer cell survival through constant adaptation without real possibility to form any sustainable system that can be stemmed for).
2. CSCs may directly derive from normal progenitor cells that may acquire tumor “stemness-like” biology through further accumulation of genetic abnormalities, including mutations and/or abnormal epigenetic modifications.
3. CSCs may directly derive from normal growing or static adult cells through genetic mutations and other mechanisms to trigger dedifferentiation. For example, by expression of hTERT, H-RasV12, and SV40LT and ST, human skin fibroblasts can be reprogrammed to have properties of CSCs [89].
4. Mathematical modeling and data analyses of thermal conditioning of glioblastoma cells suggested that stem cell-like tumor initiation cells, regardless of origin, may not be a fixed population of neoplastic cells [30]. Instead, CSC capacities such as expressions of representative markers, metastasis, oncolytic drug resistance and symmetric or asymmetric cell division may likely be a cluster of transient events occurring in a subpopulation of cancer cells when stressed or induced by environmental, epigenetic, genetic, and therapeutic impacts. Thus, the actual number of CSCs existing in a given tumor for a particular time point is determined by the optimal probability of the unilateral survival and growth of the entire tumor [90,91].
5. CSCs can emerge under varied combinatorial regimens that comprise all the aforementioned scenarios, which is a rational extrapolation that we made.

With the introduction of the fourth and fifth definitions of CSCs, data previously used as evidence to question real existence of CSCs can now be turned into valuable information to suggest an alternative concept. As an example, CSC composition ratio in different tumors might range from 0.2% to 82.5%. Using standardized limiting dilution assays, researchers uncovered that this ratio actually increased in breast cancers along their Stage I to Stage III progression. In contrast to Stage III–IV melanomas, tumorigenic cell ratios remained steadily at around 30% [92]. It has been known that CSCs in the same tumor could carry overlapping, nonoverlapping, or even varied characteristic markers [93,94].

## CSCs Are Tumor Survival Cells

In addition to the aforedescribed results, reports showed that the specific molecular mechanisms underlying commonly targeted tumor cell “stemness” are unstable. The observations of genetic instability imply a real possibility that different new parental CSC lines may continuously be produced in high-grade malignant tumors. This explains why expressions of some CSC markers are time dependent [95]. With regard to the latter point, an informative comparison case can be made by examining key profiles of ESCs versus those of ECCs that have been traditionally portrayed as opposite sides of the same coin [95].

ECCs have been identified as the “stem cells of teratocarcinomas” and as the malignant counterparts of ESCs for mammals. Unlike ESCs that are derived from the inner cell mass of early blastocyst-stage embryos, ECCs are isolated from embryonal teratocarcinomas. It is only after prolonged in vitro culture under certain regimens that some human ESCs (hESCs) start acquiring karyotypic modifications that can be observed in human ECCs (hECCs). Over the chronic incubation process, hESCs can manifest faster proliferation rate and become more maintainable in vitro. Markedly, the more transformed hESCs can form teratocarcinoma-like neoplasms in SCID mice following transplantation. Conversely, the donor hESC-derived teratocarcinoma was able to give rise to characterizable hESCs in vitro. It was therefore concluded that hESCs under particular in vitro induction conditions could develop in similar ways that hECCs do during tumorigenesis [96].

Our analysis, based on a crossdisciplinary approach of stem cell biology and developmental neurobiology, suggests that the in vitro transforming process may actually be a journey for ESCs to gradually lose their repertoire of authentic stemness biology, for which ECCs either do not own or are in severe deficiency. This postulation renders the two types of cells not at all belonging to “opposite sides of the same coin” (ie, both ESCs and ECCs possess stemness biology). The conclusion is corroborated by findings from more advanced investigations. In a study of the hECC lines, NT2/D1 and NT2/B9, which were clonally derived from a xenograft tumor of the teratocarcinoma cell line Tera-2, extensive differentiation could be induced in vitro by retinoic acid treatment [97]. This differentiation was particularly marked by the disappearance of SSEA-3 that is typically expressed by hECCs. Among the differentiated cell phenotypes, hECC-produced neuron-like cells showed morphological features of neurites and expressed tetanus toxin receptors and neurofilament proteins [97]. But these NT2/N neurons did not further mature into true neurons that could express phosphorylated neurofilament heavy (NF-H) proteins after engraftment in young adult or developmental rodent brains [98]. They nevertheless survived for >12 weeks to >1 year in rat brains under immunosuppression [98,99] and for more than 27 months in a poststroke human brain [99].

The fact that NT2/N cells showed much longer graft survival duration in the brain relative to that of freshly isolated primary neurons or neural progenitor cells indicates that they might have obtained additionally augmented individual survival efficacy, albeit reduction of neural stemness (ie, diminished ability to differentiate into mature neurons) [98–101]. Accordingly, the NT2/N neurons, not the predifferentiated NT2 progenitor cells, constitutively synthesized intracellular beta/A4 peptide, a major pathologic peptide accumulating in Alzheimer disease (AD) brains, and released it into the cell culture medium [102]. The secreted form (sAPP) of the AD amyloid beta/A4 protein precursor (APP) is a potent player in promoting neurite extension, synaptic formation, overall neurotropic support, and antiexcitotoxicity effect for neuronal cells, as well as in enhancing fibroblast growth [103–108]. Clearly, the gain of function in the ECC and ECC-derived neuron-like cells is the self-survival capability (eg, production of sAPP, enhanced grafting, etc.). Contrariwise, the loss of function for ECC and ECC-differentiated neuron-like cells is the diminished capacity of authentic stemness (eg, their inability to become mature neurons, defect in functional integration, etc.). Following this novel route of logical reasoning, cautions are deemed necessary when trying to use the NT2/N cells in vitro for investigating NSC and adult neuronal properties, modeling neuronal diseases, or discovering neuronal therapeutics [109,110]. We believe that data extrapolation without factoring the essential cell biology discrepancy could yield misleading information because changes in survival metabolic events may induce alterations of stemness marker presentations. For example, expression of CD9 gene and protein, a cell transmembrane molecule family mediating signal transductions, showed selective upregulation in human glioblastoma stem cell-like cells. CD9 silencing in three CD133+ glioblastoma cell lines triggered amelioration of cancer cell proliferation, survival, invasion, and self-renewal ability through impacting activation patterns of the Akt, MapK, and Stat3 signaling transducers, in which the signaling pathways are mainly involved in cell survival functions, which in turn resulted in expression alternations of CD133, nestin, and SOX2 [45].

Collectively, the metabolic, mitotic, and survival behavioral features as well as the overall life journey endpoints of the currently termed CSCs are vitally different from those of conventionally defined stem cells. Due to the permeating influence of stemness as an established concept that has been academically inscribed for characterizing normal primordial or tissue-specific germ cells, the use of CSC as a nomenclature may cast shadow over conscious and subconscious reasoning of investigators when they aim to tackle malignant essentials of tumor cells. Moreover, the concept of CSC certainly does not hold the original metaphorical implication of stem cells for their capacity to give rise to homeostatic and reproducible multicellular organisms, including human bodies. Therefore, establishing a new nomenclature of tumor survival cell (TSC) to replace CSC appears to be highly valuable since unilateral tumor cell survival essentials consist of endeavors of self-renewal, proliferation, limited differentiation to generate adaptive heterogeneity, migration, metastasis, immune diversity, and drug resistance.

## Potential Benefits of Adopting TSC as a New Nomenclature to Replace or Coexist with CSC

Application of TSC as an academically and scientifically further justified nomenclature may benefit the intellectual and research fields for its clarity and uniqueness that are tangibilized by the following perspectives.

1. To add special insight to the word “stem” when it is used to describe cancer cells for research and therapeutic development [110].
2. To dissect the definitive difference between TSCs and normal stem cells.
TSCs have pathologically maximized levels of individual and group survival ability and utterly perished capacity for authentic stemness biology. TSCs behave like desperados, tumor cell outlaws that act desperately to survive and appear unstoppable, but they are eventually wiped out in their self-rendered catastrophic circumstances. By contrast, ESCs or tissue-specific progenitor cells maintain a homeostatic equilibrium between its stemness biology and individual survival drive to build a sustainable and reproducible entity.
3. To reveal the lack of real stemness capability in CSCs. This will help to refocus on unilateral cell survival mechanisms when devising therapies to block cancer metastasis [111]. As *a testable hypothesis*, what is truly needed for curing the so called CSCs may likely be resurrection of authentic stemness biology and mitigation of the abnormally augmented survival biology inside tumor cells.
4. To emphasize the endpoint of the essential functions of the TSCs that is to carry out desperado-like survival behaviors (DSB). DSB as a novel term of cancer cell oncology describes the state or process of being able to live by all means available, mostly in spite of unfavorable accidents and tribulations, actually in a self-triggered perturbing, stressful, and unsustainable environment. Manifestly, tumor cell DSB do not lead to any real continuity of individual tumor cells or tumor mass as an entity. Through a single spearheaded DSB-driven relentless proliferation, growth, and metastasis, malignant tumor cells will eventually cause the imminent demise of their host organism, followed by reaching the termination of their own journey of survival. Hence, the actual use of the term TSC may further improve the fine-tuning of the direction of our reasoning in search of the oncological prerequisites of these cells in addition to providing the needed demarcation of principal differences between them and the classically entitled stem cells.

## Summary

There have been increasingly convincing experimental and clinical data that validate the genetic, epigenetic, and phenotypic heterogeneity of cells comprising malignant tumors. Although questions remain with regard to the consistency and expression levels of specific markers in a subpopulation of cancer cells behaving like stem cell-like tumor initiation cells (ie, CSCs) as well as complexity of CSC oncology, they have not been able to shake the foundation of the concept of CSCs. The current CSC models illuminate tumor generation capabilities of subpopulations of self-renewable and tumor differentiable cells that drive cancer progression via producing oncologic heterogeneity, resistance to oncolytic assaults, and the ultimate death of tumor cells due to host decease. The uncertain oncological feature and conflicting genetic signature of the CSCs (eg, CSC markers mostly are genes in charge of survival, not stemness) suggest a necessity of inaugurating a new nomenclature for the demarcation of the fundamental difference between these cells and physiological stem cells to promote cancer and stem cell research.

What emerged from our analysis is a fine but definitive line between the authentic stemness biology and the newly defined cancer cell survivology. We hereby recommend introducing the term of TSCs (*Tumor Survival Cells*). Implementation of this proposal may facilitate future work to focus on investigating key events that have gone awry in TSCs to uncover differences between the three bodies of mechanisms underlying normal stem cell biology, cancer cells, and DSB of CSCs/TSCs, respectively (Fig. 1 and Table 1). Furthermore, by weighing the characteristics of limited “developmental hierarchy” in cancer cells and full-scale “functional multipotency” of normal stem cells, researchers may explore consequences of nongenetic variability, rare clones, and clonal dynamics within a particular tumor, as well as between the tumor clones and the host microenvironment. Such undertakings may determine the oncological essentials of TSCs in terms of their endpoint of survival, defects in authentic stemness biology, and the impacts on the host. The findings will reveal crucial targets (eg, stemness defect, stemness resurrection, unchecked survival drive, etc.) for assessing risk-based patient selection for receiving a particular medical procedure and developing efficacious targeted treatment for malignant tumors [10,82,112,113].
